# Light microscopic observations of the ruminal papillae of cattle on diets with divergent forage to cereal ratios

**DOI:** 10.1016/j.animal.2022.100462

**Published:** 2022-03

**Authors:** H.J. Ferguson, H.H.C. Koh-Tan, P.E.J. Johnston, R.J. Wallace, I. Andonovic, C. Michie, C.A. McCartney, E.M. Strachan, T.J. Snelling, C.D. Harvey, W. Thomson, N.N. Jonsson

**Affiliations:** a Institute of Biodiversity, Animal Health and Comparative Medicine, University of Glasgow, Glasgow G61 1QH, UK; b School of Veterinary Medicine, University of Glasgow, Glasgow G61 1QH, UK; c Rowett Institute, Foresterhill, Ashgrove Road West, Aberdeen AB25 2ZD, UK; d Department of Electrical and Electronic Engineering, University of Strathclyde, Glasgow G1 1XW, UK; e Harbro Ltd., Turriff, Aberdeenshire AB53 4PA, UK

**Keywords:** Acidosis, Bovine, Epithelium, Histology, Rumen

## Abstract

High levels of supplementation with cereal increases production rates in cattle but can increase incidence of disease, ranging from mild indigestion to acute ruminal acidosis and death. Therefore, there is motivation to determine biological markers which can be used to identify whether animals have been, or are being fed, sufficient or excessive cereals. This study aimed to describe light microscopic findings from animals being fed diverse dietary cereal proportions and to test the performance of a novel rumen epithelial scoring system. Rumen wall tissue samples were obtained from the abattoir from 195 cattle from 11 Scottish farms and processed for histological examination. Light microscopic examination was used to characterise ruminal epithelial response to dietary challenge. Secondary objectives included describing the distribution of immune-related cells in bovine ruminal epithelium and assessing the use of a modified Elastin Martius Scarlet Blue stain (**EMSB**) for histological examination of the rumen epithelium. Cells staining positive for cluster of differentiation 3 were distributed mainly in the lower layers of the stratum basale and were found in higher densities in animals offered lower cereal proportion diets. Cells staining positive for major histocompatibility complex class 2 (**MHCII**) were most common in perivascular locations and in the junction between the lower stratum basale and the propria-submucosa. The density of MHCII positive staining cells was higher in animals on lower cereal diets. The level of supplementation with cereal was also associated with the thickness of the stratum corneum (**SCT**) and stratum granulosum (**SGT**), the integrity of the stratum corneum and sloughing of cornified cells. There were no advantages in using EMSB stain over haematoxylin and eosin (**H&E**) in this scoring system. We concluded that a scoring system that included only SCT, SGT and a measure of the loss of appearance of intercellular space allowed differentiation of groups of animals according to the level of cereal supplementation.

## Highlights

• Characteristic histological change is associated with dietary cereal proportion.
• Immune-related cells are found in higher densities in cattle on lower cereal diets.
• Cereal proportion is associated with cell layer thickness, integrity, and sloughing.
• No benefit of Elastin Martius Scarlet Blue stain use over Haematoxylin and Eosin.
• Novel scoring system can differentiate groups by approximate dietary cereal level.

## Implications

The hypothesis of this study was that a novel scoring system could classify bovine ruminal mucosa according to the diet the animal had been maintained on and its inferred risk of acidosis, as determined by approximate cereal proportion. We established that despite limited pathology in animals maintained on high cereal rations, characteristic histological changes are associated with approximate dietary cereal proportion and are identifiable via a scoring system focusing on stratum corneum and stratum granulosum thickness and a measure of the loss of appearance of intercellular space. This novel scoring system provides a reference for further investigations into acidosis-related ruminal changes.

## Introduction

Efficiently feeding cereals to ruminants requires a balance between high levels of production and animal health. High levels of cereal supplementation result in rapid growth rates but can increase the incidence of diseases, ranging from mild indigestion to acute ruminal acidosis and death (Kleen et al., 2003). Hence, there is motivation to determine biological markers that can identify whether animals have been, or are being fed, sufficient or excessive cereals. It is not currently possible to provide a repeatable and relevant classification of the microscopic features in the bovine ruminal epithelium as being common, incidental, or indicative of pathology.

It has been known for many decades that ruminal papillae change in response to dietary inputs. Well-described changes include elongation and lateral expansion (Dirksen et al., 1984), branching (Beharka et al., 1998) and proliferation of papillae (Shen et al., 2004), and thickening of the cornified layer of the epithelium (Metzler-Zebeli et al., 2013). However, histological studies of dietary effects are sparse and variability among studies means there are no comprehensive reference standards. Steele et al. (2011) found that in cattle acutely exposed to high forage and high grain diets, the thickness of the stratum corneum did not vary between diets but the thickness of the granulosum, basale and spinosum strata, together with the entire epithelium, was reduced on a high grain diet. The high grain diet was associated with increased desquamation of dead, keratinised cells from the surface of the stratum corneum. Desmosomes and zonula occludens were tighter during feeding with the high forage diet, and the stratum corneum was more intact and compact. In contrast, on high grain diets, there was a generalised loss of tissue architecture and the appearance of spaces among cells and strata, sometimes containing bacteria. Findings were consistent with a previous study by Steele et al. (2009) on a single cow. Other work, such as Liebich et al. (1987), focusing on cows in the dry period, found changes in the stratum corneum and stratum basale thickness in high fibre, low energy diets. Dieho et al. (2016), also focusing on dry cows, found that high concentrate diets during the dry period increased papillae surface area in comparison to a control diet but that the increased papillae surface area was not maintained into the subsequent lactation period. Detailed descriptions by Steele et al. (2011) provide a useful base for the development of a scoring system for ruminal pathology and health. However, the work utilised electron microscopy to examine acute responses to diet change and was limited, utilising only four animals. Electron microscopy is not well suited to a high-volume or screening approach, and although changes in epithelial architecture are clearly addressed, there is no reference to the observation of immune or inflammatory processes.

Local and systemic inflammatory responses in cattle have been reported in response to challenge with diets that are rich in cereals and concentrates (Gozho et al., 2007, Emmanuel et al., 2008, Bondzio et al., 2011; reviewed by Zebeli and Metzler-Zebeli, 2012). However, there is little literature describing the functional architecture of the immune components of the bovine foregut. Trevisi et al. (2014) attempted to address this deficiency, utilising gene expression, immunoblotting and flow cytometry of rumen tissue and content, and suggested that the innate immune components of the bovine forestomachs play an important role in modulating bovine health. Given the evidence of immune involvement in the pathology of the ruminal response to challenge diets with high levels of cereals, it might be expected that variation in the abundance and distribution of immune-related cells – those associated with innate, adaptive and cell-mediated immunity - in ruminal tissue might indicate the degree of exposure to cereals. As far as we can determine, there are no published reports that describe the distribution of any immune cells in the tissues of the rumen.

Most work to date on the light microscopic appearance of ruminal tissue has used haematoxylin and eosin (**H&E**) staining. Although H&E provides a detailed view of cellular architecture, it is not effective for defining the collagenous connective fibres in tissues, in comparison to trichrome stains such as Masson’s or Gomori Trichrome. Martius Scarlet Blue is a trichrome stain in which methyl blue is used to stain collagen, crystal scarlet stains fibrin, and red blood cells are stained yellow with picric acid. Variants of this stain are commonly used in clinical cardiology research to identify areas of clotting and fibrosis (Tyrankiewicz et al., 2016) and to study connective tissue and vascular pathology (Bulk, 1984). It should therefore be a useful stain to identify and characterise pathology in the ruminal epithelium.

The hypothesis of this study is that it is possible to use a scoring system to classify ruminal mucosa, from both beef and dairy animals, according to the type of diet the animal had been maintained on and its inferred risk of acidosis, determined by cereal proportion. The objectives were to describe the structure of the rumen epithelium, as observed using light microscopy, from animals fed diets with varying proportions of cereal and to test the performance of a novel ruminal epithelial scoring system on a large group of animals. Secondary objectives were to describe the distribution of immune cells in ruminal epithelium and to assess the use of a modified trichrome stain (Young et al., 2006) for histological examination of the rumen.

## Material and methods

### Design and animals

Rumen tissue samples were obtained from a total of 195 cattle from 11 farms on which reliable dietary information was available. Farms involved in this study were located throughout Scotland. Cattle in the study were slaughtered via humane stunning and exsanguination in accordance with UK legislation in two commercial abattoirs. All animals were slaughtered within 24 hours after their last meal, with lairage period believed to be between 12 and 18 hours for most animals. Samples were collected throughout the year, depending on the availability of animals on specific diets. The same protocol for collecting samples and initial processing was used at each location. Farms (and hence animals) were selected to provide the broadest possible range of dietary and management inputs that could be obtained in Scotland. Characteristics of the animals, farms and rations are listed in Table 1. To test a novel scoring system for the wide range of cereal to forage ratios available in Scotland, animals from the 11 farms were split into three management systems, dependent on the approximate proportion of cereal in their diet (FORAGE (0% cereal), MEDIUM CEREAL (40–60% cereal) or HIGH CEREAL (90–100% cereal)). Dietary information was determined as described in Jonsson et al. (2020) or determined via farmer questionnaire and farm visits.

**Table 1:** Characteristics of farms and animals (beef and dairy cattle) that were sampled (*n* total = 195) including farm (A–K) simplified dietary components, farm type (beef or dairy), and management system (FORAGE, MEDIUM CEREAL or HIGH CEREAL).

Farm	*n*	Type	Management system	Approximate cereal proportion (%DM)	Grass silage (%DM)	Straw (%DM)	Grass (%DM)
A	8	Beef	FORAGE	0	0	0	100
B	10	Dairy	FORAGE	0	100	0	0
C	9	Dairy	MEDIUM CEREAL	40	60	0	0
D	15	Beef	MEDIUM CEREAL	40	50	10	0
E	21	Beef	MEDIUM CEREAL	60	20	20	0
F	17	Beef	MEDIUM CEREAL	60	20	20	0
G	20	Beef	HIGH CEREAL	90	0	10	0
H	18	Beef	HIGH CEREAL	90	0	10	0
I	38	Beef	HIGH CEREAL	90	0	10	0
J	20	Beef	HIGH CEREAL	100	0	0	0
K	19	Beef	HIGH CEREAL	100	0	0	0

### Postmortem sampling

Postmortem sampling protocols were designed to ensure sampling was as close to the standard operating environment of the abattoir as possible. Sampling was carried out as described in Jonsson et al. (2020). In brief, carcasses of animals involved in the study were followed from the kill point to evisceration, with a time delay between killing and sampling of approximately 10 minutes. The rumen was incised by abattoir staff, fully emptied by hand by abattoir staff, and viewed in its entirety. A sample of approximately 8 cm by 4 cm was taken from the middle of the ventral sac – sampled due to its consistently high volume of papillae. Samples were rinsed in water to remove excess ingesta, then fixed using 10% neutral buffered formalin for 48 h. Following this, they were removed and stored in 1 × phosphate-buffered saline until they could be embedded in paraffin cassettes for histology and immunohistochemistry.

### Histology and immunohistochemistry

Histology processing and staining were carried out as described in Jonsson et al. (2020). In brief, samples were cut into 1–3 sections, dependent on size, and embedded in paraffin before being processed on a microtome at 3 μm and placed onto slides. H&E and Elastin Martius Scarlet Blue (**EMSB**) staining was then carried out manually by HJF, as described in Jonsson et al. (2020). Immunohistochemistry staining was carried out by Veterinary Diagnostic Services at the University of Glasgow’s School of Veterinary Medicine, as described in Jonsson et al. (2020). In brief, five slides were stained per sample for all samples: major histocompatibility complex class 2 (**MHCll**), myeloperoxidase, cluster of differentiation 3 (**CD3**), negative controls for rabbit and mouse immunoglobulins, using an Autostainer (Dako).

### Examination, image capture, storage and analysis

All slides were initially scanned using an Olympus CX41 microscope. Images of typical and atypical examples of all features of interest were captured using GXCam software (GTvision, UK), calibrated as recommended by the manufacturer, using ×4, ×10 and ×40 graticules. In each case, two images from each of two randomly chosen papillae, ensuring the papillae were as complete as possible and the field was free of artefacts, were examined from each slide. There was no minimum size of papillae determined, and measurement points were randomly chosen across the viewing field. All slides were examined and scored by one operator for each set of samples (HJF). However, during the development of the scoring system used, the team utilised a multi-header microscope, ensuring agreement on the scoring system by scoring multiple slides together and ensuring correlation of their ranking. Criteria for scoring are summarised in Table 2 and described in full in Supplementary Table S1. All stained slides were of a quality to apply the scoring system fully.

**Table 2:** Summary of variables measured in the standardised scoring system applied to bovine rumen epithelium from beef and dairy cattle. See Supplementary Table S1 for full scoring system.

Name & Abbreviation	Definition and How Measured	Levels
SC thickness (SCT)	Mean of 5 measurements in µm across the SC over 2 fields.	NA
SG thickness (SGT^1^)	Mean of 5 measurements in µm across the SG, SB and SS over 2 fields.	NA
Clefting and complexity (CLEFT)	Presence of clefts, buds, branches along the papillae	1–3
Integrity of the SC (SCINT)	The extent to which the SC forms a complete, uninterrupted layer over the papillae.	1–5
Microabscess (MICRO)	Presence of microabscesses in any papillae observed.	0,1
Cytoplasmic swelling (SWELL)	Loss of normal appearance of intercellular space.	1–3
Cytoplasmic swelling score (SWELLSCORE)	Sum of SWELL results from two slides.	2–6
Perinuclear vacuolation (VAC)	Presence of perinuclear vacuoles in the SB and SS.	0,1
Intracellular vacuolation score (VACSCORE)	Sum of binary results from two slides.	0–2
Sloughing (SLOUGH)	Retention or partial retention of sheaths of SC on papillae	1–3
Vessel diameter (VASCD)	The diameter of the single largest vessel in each of two papillae.	NA
CD3^+^ cell (CD3^+^)	Count of the total number of CD3 positive cells in a single image.	NA
MHCII^+^ cell (MHCII^+^)	Count of the total number of MHCII positive cells in a single image.	NA

Abbreviations: SC = stratum corneum; SG = stratum granulosum; SB = stratum basale; SS = stratum spinosum; NA = not applicable; CD3 = cluster of differentiation 3; MHCII = major histocompatibility complex class 2.

1 As cell layer differentiation is not always clear, SGT measurement should include SB and SS thickness.

### Statistical analysis

Data were analysed using R (Version 4.0.3, R Core Team, 2018). Continuous data were checked for normality by examination of histograms and the Shapiro-Wilk test. Data that deviated significantly from a normal distribution were natural log-transformed and re-examined. In all cases, natural log-transformation resulted in a significant improvement in the conformity with a normal distribution and was considered to provide a sufficiently close approximation to a normal distribution to enable parametric statistical analysis. Where there was any doubt, non-parametric methods were used. The effect of farm of origin on each transformed variable was estimated using one-way ANOVA, as was the effect of management system (FORAGE, MEDIUM or HIGH CEREAL). Where data deviated significantly from a normal distribution, the Kruskal-Wallis test was used to quantify effects. For factor analysis and principal component analysis, each observation in each variable was standardised by dividing the observation by the overall median for that variable. Factor analysis was carried out without rotation of components. To identify the factors to retain and the important variables loading the retained factors, principal component analysis was undertaken, supplemented with parallel analysis using the “paran” package in R (Version 1.5.2), set to 5000 iterations. Three sets of standardised variables were then selected for inclusion in preliminary scoring systems. Scores were checked for normality and then the effects of farm and management system on each of the scores evaluated by ANOVA. Four scoring systems were selected to apply to the dataset.

## Results

### Tissue architecture and description of scoring system features

**Fig. 1:**
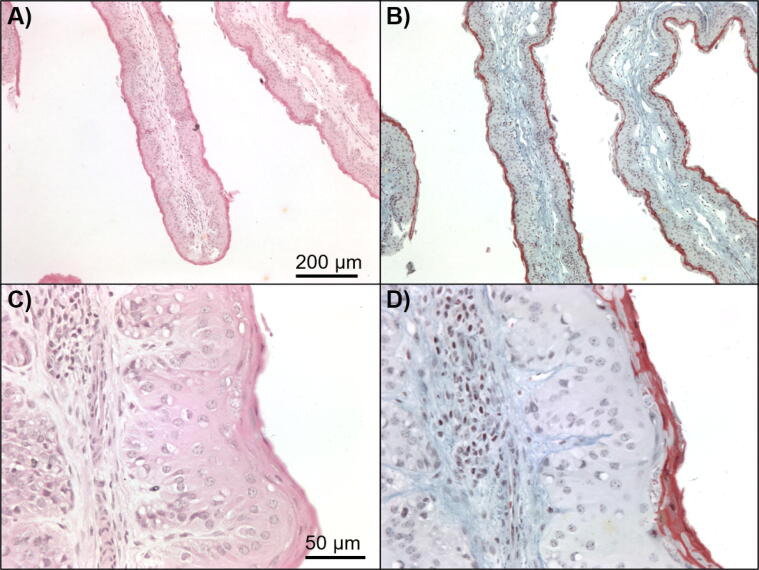
Representative section of bovine rumen papilla stained with (A–B) Haematoxylin and Eosin (H&E) or (C–D) Elastin Martius Scarlet Blue (EMSB) at ×10 and ×40 magnifications, respectively.

**Fig. 2:**
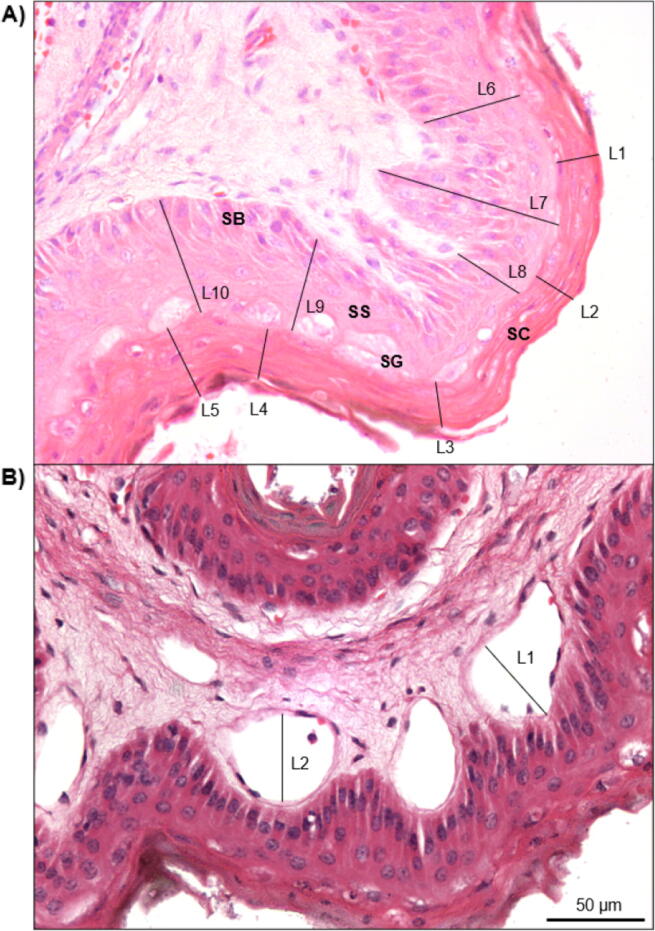
Bovine rumen papillae showing measurements and derivation of (A) stratum corneum thickness (SCT), shown in lines 1–5 (L1–L5), and stratum granulosum thickness (SGT), shown in L6–L10, both at ×40 magnification; and (B) vessel diameter (VASCD) at ×40 magnification on sections stained with haematoxylin and eosin (H&E) (L1–L2).

**Fig. 3:**
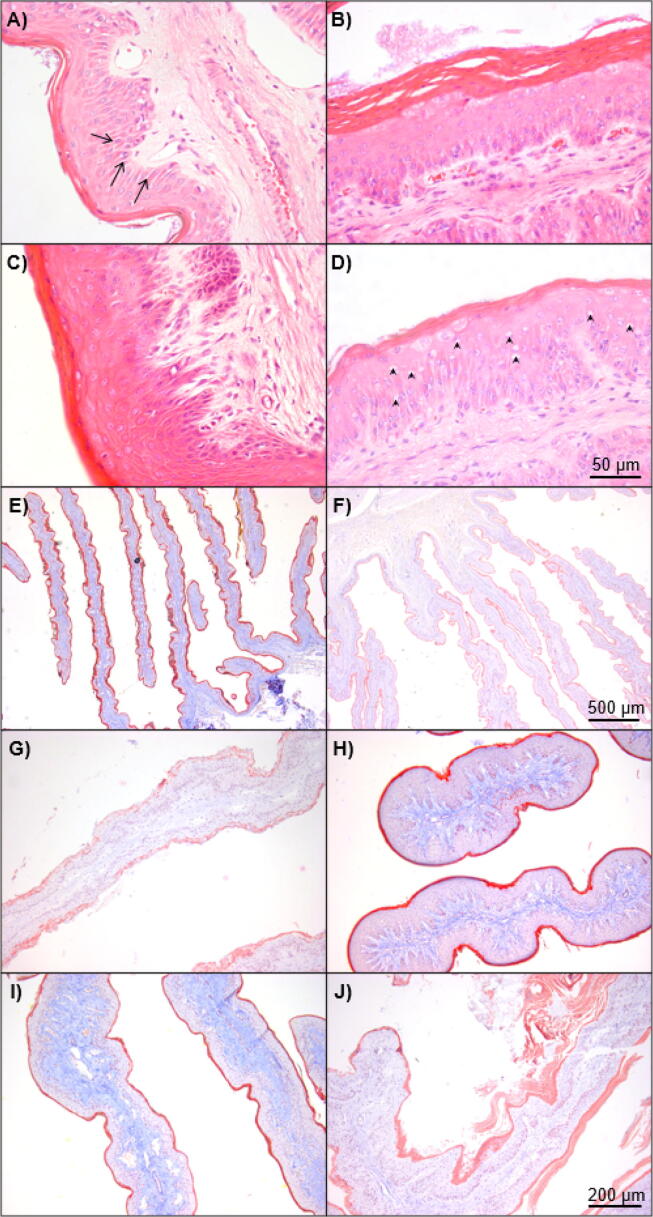
Representative examples of categorical data type shown in bovine rumen papillae. (A) Low and (B) high cytoplasmic swell score where the loss of cellular definition is evident, with arrows pointing to intercellular spaces. (C) Low and (D) high perinuclear vacuolation score with arrowheads pointing to perinuclear vacuoles. (E) Low and (F) high clefting scores; (G) Low and (H) high stratum corneum integrity scores; and (I) Low and (J) high slough scores. Magnification at ×4 (E–F), ×10 (G–J) and ×40 (A–D) with the scale bars on the last image of each magnification.

**Fig. 4:**
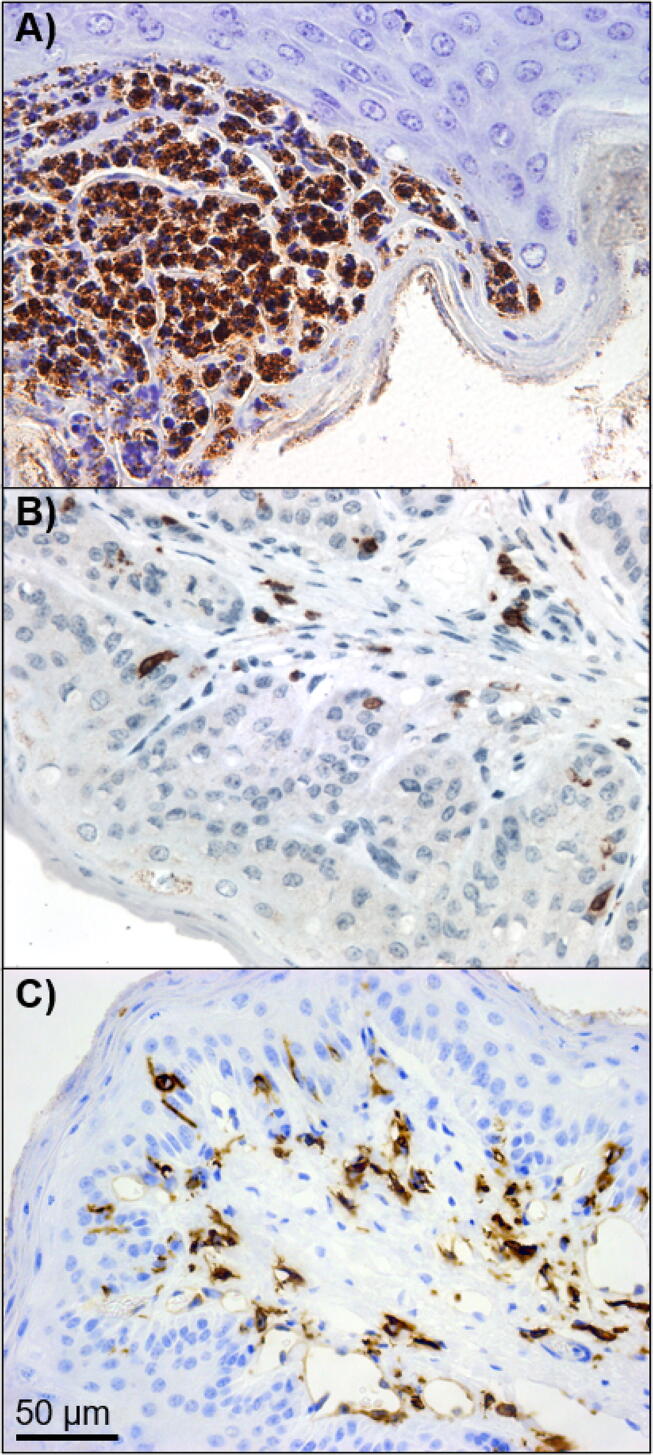
Representative immunohistochemical staining for myeloperoxidase, cluster of differentiation 3 (CD3) and major histocompatibility complex 2 (MHCII) in bovine rumen papillae. (A) Myeloperoxidase-stained microabscess between stratum corneum and granulosum. (B) CD3-stained papilla showing the typical distribution of CD3 positive staining cells, mainly at the junction between the lower stratum basale and the propria-submucosa. (C) MHCII-stained papilla showing the typical, predominantly perivascular, distribution of MHCII positive staining cells, particularly in the lower stratum basale and the propria-submucosa. Magnification at ×40.

Fig. 1 shows representative sections of papillae stained with H&E (A–B) and EMSB (C–D) at low magnification. High magnification images that indicate measurements and derivation of continuous variables reported in this study are shown in Fig. 2. Representative examples of high and low values for each of the categorical features included as variables in the scoring system, as described in Table 2, are shown in Fig. 3(A–J). Representative examples of myeloperoxidase, CD3 positive and MHCII positive staining of ruminal papillae are shown in Fig. 4A–C, respectively. Other than highlighting the presence of microabscesses (also easily detected using H&E staining), myeloperoxidase staining was not informative, and no myeloperoxidase-stained cells were counted outside microabscesses. Microabscesses, focal aggregations of neutrophils in the stratum corneum, were present in many samples from animals from all management systems. No pathology, other than microabscesses, was noticed on histological examination of the ruminal epithelium of any of the animals in this study.

### Continuous variables

The results of histological examinations of slides from all animals, means and standard deviations of all the continuous variables are listed in Table 3. Box and whisker plots of each of the measured variables according to the farm and management system (FORAGE, MEDIUM CEREAL, HIGH CEREAL) are shown in Supplementary Fig. S1. Farm had a significant effect on all continuous variables. System had a significant effect on all continuous variables, except **VASCD** (vessel diameter). Stratum corneum thickness (**SCT**) and stratum granulosum thickness (**SGT**) were highest in the HIGH CEREAL group, and lowest in the FORAGE group and correlated positively as expected (*r* = 0.45, *P* < 0.05). The stratum corneum was approximately four cells thick in most areas but ranged from two cells to over 10 cells thick. However, some animals showed extreme thickening of the stratum corneum, with more than 10 cell layers localised to individual papillae or parts of papillae. In some animals, the cornified epithelium was adherent to the underlying layers, whereas in others, the thickened stratum corneum could be seen sloughing off. Loss of distinct cellular structure in the stratum basale and granulosum was evident in the animals in the HIGH CEREAL group, as was a reduction in cleft formation. VASCD differed among farms (*P* < 0.0001) and was generally highest in animals in the HIGH CEREAL group, though not significantly so (*P* > 0.05). The abundance of CD3 positive staining cells was different by farm and management system (*P* < 0.0001), generally being higher in animals on the FORAGE diets in comparison to the two CEREAL groups. The count of MHCII positive staining cells was different by farm and management system (*P* < 0.0001) and was again higher in the FORAGE group in comparison to the two CEREAL groups.

**Table 3:** Summary of bovine rumen histological observations on 195 samples (beef and dairy cattle) according to their management system (FORAGE, MEDIUM CEREAL or HIGH CEREAL) and farm (A–K).

		FORAGE	MEDIUM CEREAL	HIGH CEREAL		
Variable (±SD)		(*n* = 18)	(*n* = 62)	(*n* = 115)	System *P-*value	Farm *P*-value
Continuous Variables^1^						
SCT (µm)		3.72 ± 0.88	6.15 ± 2.63	9.03 ± 6.07	<0.0001	<0.0001
SGT (µm)		25.74 ± 7.69	34.63 ± 7.63	41.05 ± 19.44	<0.0001	<0.0001
VASCD (µm)		23.07 ± 6.28	21.69 ± 7.26	27.26 ± 17.85	0.236	<0.0001
CD3^+^		61.44 ± 16.61	48.61 ± 17.22	55.84 ± 26.35	<0.0001	<0.0001
MHCII^+^		70.33 ± 16.31	55.84 ± 26.35	45.66 ± 18.97	<0.0001	<0.0001
Categorical Variables^2^						
CLEFT					0.027	<0.0001
	CLEFT 1	1	19	31		
	CLEFT 2	4	18	36		
	CLEFT 3	13	25	48		
SWELL					0.007	<0.0001
	SWELL 2	2	2	1		
	SWELL 3	4	6	13		
	SWELL 4	7	35	45		
	SWELL 5	4	17	33		
	SWELL 6	1	2	23		
VAC					0.13	<0.0001
	VAC 0	4	8	13		
	VAC 1	9	25	42		
	VAC 2	5	29	60		
SCINT					<0.001	<0.0001
	SCINT 1	5	9	7		
	SCINT 2	5	10	12		
	SCINT 3	1	17	34		
	SCINT 4	6	18	14		
	SCINT 5	1	8	48		
SLOUGH					<0.0001	<0.0001
	SLOUGH 1	4	41	33		
	SLOUGH 2	6	16	58		
	SLOUGH 3	8	5	24		
MICRO					0.17	<0.0001
	MICRO 0	11	27	66		
	MICRO 1	7	35	49		

Abbreviations: SCT = stratum corneum thickness; SGT = stratum granulosum thickness; VASCD = vessel diameter; CD3 = cluster of differentiation 3; CD3+ = count of CD3 positive cells; MHCII = major histocompatibility complex class 2; MHCII+ = count of MHCII positive cells; CLEFT = clefting and complexity score; SWELL = cytoplasmic swelling; VAC = presence of perinuclear vacuoles in stratum basale and stratum spinosum; SCINT = integrity of the stratum corneum; SLOUGH = retention or partial retention of stratum corneum; MICRO = presence or absence of microabscesses.

1 Continuous data results expressed as mean ± SD for the group.

2 Categorical data expressed as the count of each factor level.

### Categorical variables

The results of histological examinations of slides from all animals and counts of all categorical variables are listed in Table 3. Bar charts of the frequencies of each of the levels of the categorical variables by farm and by management system are shown in Supplementary Fig. S2. The integrity of the stratum corneum and the predisposition to sloughing of the stratum corneum were affected by the approximate level of cereal, with higher scores for loss of integrity being seen more frequently with the HIGH CEREAL system. However, although the effect of management system was significant, there was inconsistency among farms in each system. The frequency of observation of high scores for sloughed cells from the stratum corneum, despite differing among levels of cereal, was not consistently associated with HIGH or MEDIUM cereal diets. The frequency of samples with high scores for epithelial cytoplasmic swelling showed a consistent and significant tendency to be higher with HIGH CEREAL diets.

### Principle component analysis

The eigenvalues obtained from Horn’s parallel analysis for factor retention are listed in Supplementary Table S2. The analysis suggested that three factors should be retained (factors for which adjusted eigenvalues > eigenvalues of simulated data). The results of the factor analysis, selecting three factors, are shown in Supplementary Fig. S3 and the loadings of each of the variables for the three retained factors are shown in Supplementary Table S3. Variables were included in the initial model if they had a loading with an absolute value ≥0.3. They were included with sign derived from their loading on the first factor. For example, loadings on Factor 1 were SCT = 0.7, CD3 = −0.79, so SCT was assigned a positive value and CD3 was assigned a negative value. Three further versions of the score were compiled by progressively excluding either variables with lower loading or low uniqueness values. Scores are shown in Table 4, together with the results of one-way ANOVA for each score against the factors of farm and management system. Boxplots of the scores by farm and management system are shown in Supplementary Fig. S4. For each of the four scoring systems, the management system had a significant effect, the greatest effect being noted on Score 4 (*P* < 0.0001). Supplementary Fig. S5A shows each of the four scoring systems, categorised by the approximate level of cereal supplementation, showing that Score 4 provided the best discrimination among cereal inputs. Supplementary Fig. S5B shows Score 4 applied to all animals, where A–K represent farms and individual animal scores are plotted along the x-axis. Within level of cereal, scores varied widely and although the mean value for a group might be a reasonable predictor of the approximate cereal level, the score is not a useful test to be applied to a single sample. The relationships between the histological Score 4 and the levels of straw and grass silage inclusion are shown in Supplementary Fig. S6. Neither of these factors were associated with the score (*P* > 0.05).

**Table 4:** Scores for evaluating histological change in bovine rumen samples from 195 animals (beef and dairy cattle), variables included and the association of each score with farm (A–K) and management system (FORAGE, MEDIUM CEREAL or HIGH CEREAL), based on one-way ANOVA.

	Variables Included					
	Positive sign	Negative sign	Farm *F-*value	Farm *P-*value	System *F-*value	System *P*-value
Score 1	SCT, SGT, SCINT, VACSCORE, SLOUGH, SWELLSCORE	CLEFT, VASCD, MICRO, MHCII^+^	9.749	<0.001	20.13	<0.001
Score 2	SCT, SGT, SCINT, VACSCORE, SLOUGH, SWELLSCORE	CLEFT, MICRO	9.572	<0.001	17.66	<0.001
Score 3	SCT, SGT, SCINT, VACSCORE, SWELLSCORE	CLEFT	23.28	<0.001	38.2	<0.001
Score 4	SCT, SGT, SWELLSCORE		20.83	<0.001	60.98	<0.001

Abbreviations: SCT = stratum corneum thickness; SGT = stratum granulosum thickness; SCINT = integrity of the stratum corneum; VACSCORE = intracellular vacuolation score; SLOUGH = retention or partial retention of stratum corneum; SWELLSCORE = swell (loss of appearance of intercellular space) score; CLEFT = clefting and complexity score; VASCD = vessel diameter; MICRO = presence of microabscesses; MHCII+ = count of major histocompatibility complex class 2 (MHCII) positive cells. Variables fully defined in Table 2.

## Discussion

This histological scoring system enables a reasonable prediction of the approximate level of cereal feeding of groups of animals, but the wide variation in among-animal histological observations within farm or diet precludes accurate prediction based on a single animal, except at the most extreme values of the cereal feeding spectrum. The three most robust or consistent components of the scoring system are the thickness of the stratum corneum and granulosum and the degree of cytoplasmic swelling of epithelial cells. These findings were highly consistent, increasing as the approximate proportion of cereals in the diet increased. Studies that cite thickening of the ruminal epithelium in the literature have sometimes referred to parakeratosis or hyperkeratosis (Bull et al., 1965, Hinders and Owen, 1965), however, the use of the term parakeratosis (retention of the nuclei in the cornified layer) appears to be inconsistent and the extent to which parakeratosis is a normal finding requires clarification. Similarly, exactly how hyperkeratosis is defined in bovine rumen requires clarification. The consistent finding from our study of increased stratum corneum and granulosum thickness with increasing dietary cereal proportion is consistent with some studies (Nocek et al., 1984, Metzler-Zebeli et al., 2013). However, they contrast with Steele et al., 2009, Steele et al., 2011, and 2012), in which the exposure to high cereal grain was more acute than in the animals on high cereal in our study. All animals in our study fed high cereal proportions had at least 90–120 days to adapt to their respective diets before sampling. This critical difference is reflected in the difference between the responses of the short-chain fatty acid (**SCFA**) concentrations in Steele’s work in comparison with ours (partially overlapping group of 119 cattle, described in Jonsson et al., 2020). In the high cereal cattle in our previous study, there was a negative relationship between cereals and SCFA concentrations, whereas in Steele’s study (2011), the relationship is positive. We interpret the increase in thickness, in association with a reduction in concentration of SCFAs, as a long-term adaptive response to challenge with cereals, following an initial acute increase in SCFAs (Metzler-Zebeli et al., 2013, Jonsson et al., 2020). Other studies which have shown a clear difference in short- and long-term epithelial responses include Dieho et al. (2016) who noted that rapid increase of concentrate resulted in increased papillae surface area when compared to a gradual increase. Despite this initial increase, they noted that overall microscopic morphology was not affected and that the surface area increase was not maintained.

The detailed descriptions of cell morphology and tissue architecture in light and electron microscopy from Steele et al. (2011) differ from ours. Steele et al. (2011) described a higher level of epithelial organisation and tighter cellular junctions in animals on forage-based diets. Electron microscopy images clearly show increased separation of cells in animals on the high grain diets. Bacteria are clearly visible in these spaces, and cellular junctions are clearly disrupted. In our study, cellular swelling was considered present when there was a loss of observable space between cells and loss of the characteristic appearance of tight junctions among the cells of the stratums basale, spinosum and granulosum and it increased with increasing cereal proportion of the diet. Unfortunately, as we did not do electron microscopy studies, it is difficult to directly relate our findings with those of Steele et al. Vacuolation of cells in the stratum granulosum was an inconsistent finding in our study and not related to cereal in the diet. Perinuclear vacuolation observed within cells of the stratum granulosum in cattle on high cereal diets is broadly consistent with observations by Dobson et al. (1956) in sheep, in which it was noted that at the edge of the stratum corneum, swollen misshapen cells were common, along with variable interstitial spaces and vesiculation in the stratum granulosum. However, the diet on which the sheep were maintained in that study was not stated and so observations cannot be linked with cereal intake.

Although the immune markers chosen in this study are not representative of the entire bovine immune or inflammatory response, they were chosen to give an overview of some of the components likely to be involved in a response to dietary challenge. Myeloperoxidase was chosen to highlight neutrophils as a marker of innate immunity or inflammation, and MHCII together with CD3 were chosen as indicators of adaptive immune responses. Cells staining positive for MHCII and CD3 were found mainly in the junction between the lower stratum basale and the propria-submucosa of the rumen papillae. The densities of MHCII and CD3 positive staining cells tended to decrease as cereal feeding levels increased, with the lowest densities of CD3 positive staining cells being found in the MEDIUM cereal-fed beef groups and the highest levels in the FORAGE group. The lowest levels of MHCII positive staining cells were found in the HIGH cereal-fed group with the highest densities also in the FORAGE group. The EMSB stain enabled clear visualisation of the integrity of the stratum corneum, but none of the observations included in the scoring systems were dependent on it. Its value did not justify the considerable additional work involved, although it is possible that we have overlooked potentially useful variation in staining patterns. Myeloperoxidase stain was not informative in this study. Overall, H&E staining seems to be sufficient to provide reasonable discrimination among animals on these diets.

Microabscesses were found in many of the animals in this present study but are scarcely reported in the literature. It is unclear what causes microabscesses; the authors of one study on reindeer calves fed on diverse diets suggested that ruminal microabscesses are caused by plant particles penetrating the rumen epithelium (Josefsen et al., 1997), but did not seem to indicate an inferior diet nor influence the health of the animal. However, Schilcher et al. (2013) proposed that microabscesses in wild ruminants may be related to diets high in cereals and associated them with subacute ruminal acidosis.

There was a very strong effect of farm of origin on the histological variables examined in this study. Because of the unbalanced factorial design that is a feature of observational studies such as this, it is difficult to definitively partition variance among the input factors. However, the approximate level of cereal supplementation was a very consistent and strong effect of a very similar magnitude to the effect of farm. In any consideration of proportional dietary inputs, cereal and roughage are always negatively correlated, and so a similar but inverted relationship with histological variables would be expected for grass silage and straw, as was seen for cereal. Although such a relationship was noted in the present study, it was orders of magnitude weaker for both grass silage and straw than the relationships with cereal supplementation, being weakest with straw. A weak relationship between histological findings and the level of straw inclusion was expected because the information used in this study regarding the inclusion of straw was quite weak. For finishing beef cattle in Scotland, straw may be included in a mixed ration provided to cattle or it may be provided *ad libitum* in ring-feeders, or in some cases, it is only available to cattle from the bedding. The potential lack of distinction of these possibilities could have led to inaccurate recording of the approximate amount of straw fed. Principal component analysis was intentionally undertaken on the dependent variables without consideration of any of the input variables, with the objective of determining how many potential underlying factors contributed to the observed variation in histology. It suggested that three factors should be retained in the model, but the second and third factors had very low adjusted eigenvalues in the parallel analysis. Given this and the strong effect of cereal proportion on the individual variables and on the scores that were based on principle components, it seems reasonable to ascribe most of the variation in histological findings to the single factor of the level of cereal proportion.

## Conclusions

This paper aimed to describe the light microscopy findings from animals with diverse management and nutritional backgrounds, and to test the performance of a novel rumen epithelial scoring system on animals from a range of management systems. Secondary objectives were to describe the distribution of immune-related cells in ruminal epithelium and to assess the use of EMSB stain for histological examination of the rumen. We have shown that the scoring system could differentiate groups of animals according to the approximate level of cereal in their diet, although the high level of individual variation within farms or diets precludes discrimination of diet based on a single observation. We found that CD3 positive staining cells were distributed mainly in the lower layers of the stratum basale, and their density was higher in animals on lower cereal diets. Cells staining MHCII positive were most common in perivascular locations, also in the junction between the lower stratum basale and the propria-submucosa. The density of MHCII positive staining cells was also higher in animals on lower cereal diets. We found H&E stain to be sufficient to discriminate among diets.

## Supplementary material

Supplementary data to this article can be found online at https://doi.org/10.1016/j.animal.2022.100462.

## Ethics approval

Not applicable.

## Data and model availability statement

The full dataset which supports findings from this study is freely available from the University of Glasgow Enlighten repository at: https://doi.org/10.5525/gla.researchdata.710.

## Author ORCIDs

**H.J. Ferguson:**https://orcid.org/0000-0003-1487-7076.

**N.N. Jonsson:**https://orcid.org/0000-0003-3245-9783.

**H.H.C. Koh-Tan:**https://orcid.org/0000-0001-5842-6491.

**P.E.J. Johnston:**https://orcid.org/0000-0002-2743-4525.

**R.J. Wallace:**https://orcid.org/0000-0003-2691-1585.

**I. Andonovic:**https://orcid.org/0000-0001-9093-5245.

**C. Michie:**https://orcid.org/0000-0001-5132-4572.

**C.A. McCartney:**https://orcid.org/0000-0001-5845-8151.

**T.J. Snelling:**https://orcid.org/0000-0002-8952-8826.

## Author contributions

**H.J. Ferguson:** Methodology, Investigation, Formal Analysis, Writing - Original draft, Writing – Review and editing.

**H.H.C. Koh-Tan:** Methodology, Investigation, Project Administration, Writing - Original draft, Writing – Review and editing.

**P.E.J. Johnston:** Writing – Review and editing.

**R.J. Wallace:** Conceptualisation, Funding Acquisition, Methodology, Investigation.

**I. Andonovic:** Conceptualisation, Funding Acquisition.

**C. Michie:** Conceptualisation, Funding Acquisition.

**C.A. McCartney:** Methodology, Investigation.

**E.M. Strachan:** Investigation.

**T.J. Snelling:** Investigation, Writing – Review and editing.

**C.D. Harvey:** Investigation.

**W. Thomson:** Conceptualisation, Funding Acquisition, Methodology.

**N.N. Jonsson:** Conceptualisation, Funding Acquisition, Methodology, Formal Analysis, Investigation, Visualisation, Writing - Original draft, Writing – Review and editing.

## Declaration of interest

None.
