# Supplementary material for: Light microscopic observations of the ruminal papillae of cattle on diets with divergent forage to cereal ratios
Source: Animal. 2022 Mar;16(3):None. doi: 10.1016/j.animal.2022.100462 (PMC8934251; doi:10.1016/j.animal.2022.100462)

Supplementary Material – Online Publication Only

Animal

Light microscopic observations of the ruminal papillae of cattle on diets with divergent forage to cereal ratios

H. J. Ferguson a,1, H. H. C. Koh-Tana,, P. E. J. Johnstonb, R. J. Wallacec, I. Andonovic^d^, C. Michie^d^, C. A. McCartney^c^, E. M. Strachan^a^, T. J. Snelling^c^, C. D. Harvey^e^, W. Thomson^e^, N. N. Jonsson^a^

Supplementary Table S1: Detailed description of the variables in a standardised histological scoring system applied to bovine rumen epithelium, including variable name and abbreviation, type, definition and how it was measured, levels and level definitions.

| Name & Abbreviation | Variable Type | Definition and How Measured | Levels | Level Definitions |
| --- | --- | --- | --- | --- |
| SC thickness (SCT) | Continuous | Mean of 5 measurements in µm across the SC over 2 fields, using x40 magnification and H&E stain. | NA | NA |
| SG thickness (SGT) | Continuous | Mean of 5 measurements in µm across the SG, SB and SS over 2 fields, using x40 magnification and H&E stain. | NA | NA |
| Clefting and complexity (CLEFT) | Ordinal categorical | Presence of clefts, buds, branches along the papillae, using x4 magnification and EMSB stain. | 1-3 | 1 – All or almost all papillae are simple with no clefts, buds, branches  2 – Minority of papillae show some degree of cleft, bud or branch formation  3 – Majority of papillae show some degree of cleft, bud or branch formation |
| Integrity of the stratum corneum (SCINT) | Ordinal categorical | The extent to which the SC forms a complete, uninterrupted layer over the papillae, using x10 magnification and EMSB stain. | 1-5 | 1 – All or almost all papillae show intact SC as a single, scarlet band on EMSB stain  2 – Minority of papillae show vacuolation in the SC, but the SC remains clearly identifiable as an intact, single, scarlet band on EMSB stain  3 – Majority of papillae show vacuolation in the SC, but the SC remains clearly identifiable as an intact, single, scarlet band on EMSB stain  4 – Minority of papillae show vacuolation in the SC and interruptions to the integrity of the SC, which is not clearly identifiable as an intact, single, scarlet band on EMSB stain  5 – Majority of papillae show vacuolation in the SC and interruptions to the integrity of the SC, which is not clearly identifiable as an intact, single, scarlet band on EMSB stain. |
| Microabscess (MICRO) | Binary | Presence of microabscesses in any papillae observed using x10 magnification and myeloperoxidase stain | 0,1 | 0 – No microabscesses seen  1 – Microabscesses of any number seen |
| Cytoplasmic swelling (SWELL) | Ordinal categorical | Loss of normal appearance of intercellular space (evidenced as observable space between cells and loss of the characteristic appearance of tight junctions among the cells of the SB, SS and SG, using x40 magnification and H&E stain. | 1-3 | 1 – Intercellular spaces clearly visible among the majority of cells in all layers  2 – Intercellular spaces clearly visible among some cells but not consistently among layers  3 – Intercellular spaces not visible at all |
| Cytoplasmic swelling score (SWELLSCORE) | Ordinal categorical | Loss of normal appearance of intercellular space (evidenced as observable space between cells and loss of the characteristic appearance of tight junctions among the cells of the SB, SS and SG, using x40 magnification and H&E stain. | 2-6 | Sum of SWELL results from two slides |
| Perinuclear vacuolation (VAC) | Binary | Presence of perinuclear vacuoles in the SB and SS using x40 magnification and H&E stain. | 0,1 | 0 – No vacuolated cells seen  1 – Vacuolated cells noted |
| Intracellular vacuolation score (VACSCORE) | Ordinal categorical | Presence of intracellular vacuoles in the SB and SS using x40 magnification and H&E stain. | 0-2 | Sum of binary results from two slides, each treated as per VAC |
| Sloughing (SLOUGH) | Ordinal categorical | Retention or partial retention of sheaths of SC on papillae, observed using x10 magnification and EMSB stain | 1-3 | 1 – No papillae show evidence of retained sheaths of cornified cells  2 – Few or occasional papillae show evidence of retained sheaths of cornified cells  3 – Many papillae show evidence of retained sheaths of cornified cells |
| Vessel diameter (VASCD) | Continuous | The diameter of the single largest vessel in each of two papillae using x40 magnification and H&E stain. | NA | NA |
| CD3^+^ cell (CD3^+^) | Count | Count of the total number of CD3^+^ cells in a single image taken at x40 magnification. | NA | NA |
| MHCII^+^ cell (MHCII^+^) | Count | Count of the total number of MHCII^+^ cells in a single image taken at x40 magnification. | NA | NA |

Abbreviations: SC = stratum corneum; SG = stratum granulosum; SB = stratum basale; SS = stratum spinosum; H&E = Haematoxylin and Eosin stain; EMSB = Elastin Martius Scarlet Blue stain; CD3 = cluster of differentiation 3; MHII = major histocompatibility complex class 2; CD3+ = CD3 positive staining cells; MHII+ = MHCII positive staining cells; NA = not applicable.

Supplementary Table S2: Results of Horn's parallel analysis (PA), using data from all animals (dairy and beef cattle, n = 195), for factor retention in the scoring systems with 5000 iterations, using the mean estimate.

| Factor | Adjusted  Eigenvalue | Unadjusted  Eigenvalue | Estimated  Bias |
| --- | --- | --- | --- |
| 1 | 1.62991 | 2.093927 | 0.463935 |
| 2 | 0.242963 | 0.581202 | 0.338238 |
| 3 | 0.108818 | 0.355051 | 0.246232 |
| 4 | -0.12768 | 0.153764 | 0.166532 |
| 5 | -0.079475 | 0.015953 | 0.095428 |
| 6 | -0.029538 | -0.00039 | 0.29146 |
| 7 | -0.45905 | -0.07899 | -0.03309 |
| 8 | -0.048898 | -0.14126 | -0.09236 |
| 9 | -0.059759 | -0.21092 | -0.15116 |
| 10 | -0.962739 | -1.17515 | -0.21241 |
| 11 | -2.417772 | -2.70061 | -0.28284 |

Supplementary Table S3: Loadings and uniqueness estimate for each of the variables in a three-factor model, using data from all animals (dairy and beef cattle, n = 195), testing the hypothesis that 3 factors are sufficient. The chi-square statistic is 26.94 on 25 df, P = 0.36.

| Variable | Factor 1 | Factor 2 | Factor 3 | Uniqueness |
| --- | --- | --- | --- | --- |
| SCT | 0.70 | 0.35 | 0.32 | 0.29 |
| SGT | 0.80 | 0.60 |  | 0.00 |
| CD3^+^ | -0.79 | 0.61 |  | 0.00 |
| MHCII^+^ | -0.52 |  |  | 0.68 |
| VASCD | 0.30 |  |  | 0.81 |
| SWELLSCORE |  |  |  | 0.91 |
| CLEFT |  |  |  | 0.97 |
| SCINT |  |  |  | 0.88 |
| SLOUGH |  |  |  | 0.77 |
| MICRO |  |  |  | 0.94 |
| SS loadings | 2.18 | 1.06 | 0.57 |  |
| Proportion Variation | 0.20 | 0.10 | 0.05 |  |
| Cumulative Variation | 0.20 | 0.29 | 0.35 |  |

Abbreviations: SCT = stratum corneum thickness; SGT = stratum granulosum thickness; CD3 = cluster of differentiation 3; CD3^+^ = Count of CD3 positive staining cells; MHCII = major histocompatibility complex class 2; MHII^+^ = Count of MHCII positive staining cells; VASCD = vessel diameter; SWELLSCORE = Count of cytoplasmic swelling score; CLEFT = clefting and complexity score; SCINT = integrity of the stratum corneum; SLOUGH = retention or partial retention of stratum corneum; MICRO = Presence of microabscesses; SS = sum of squared loadings.


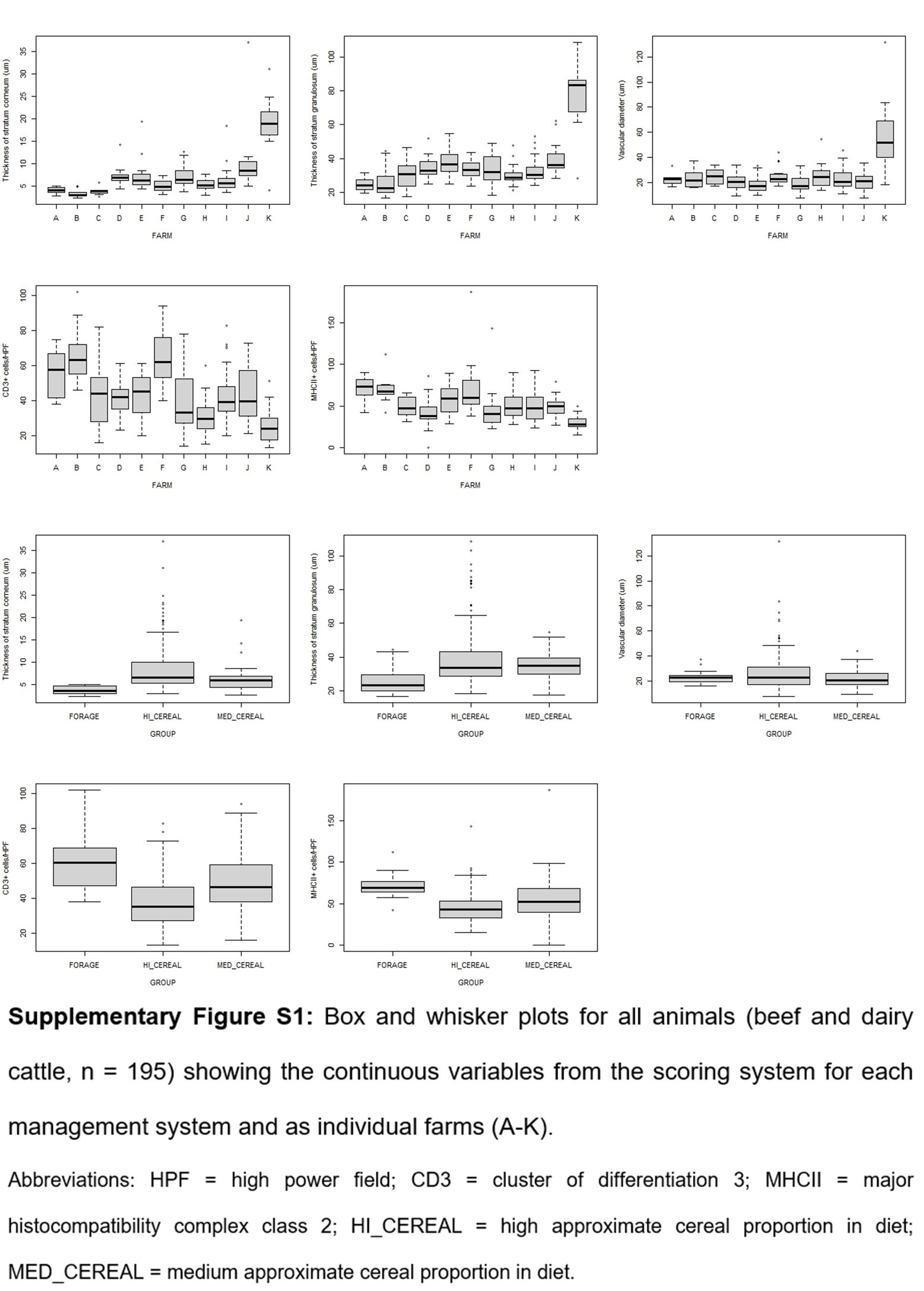


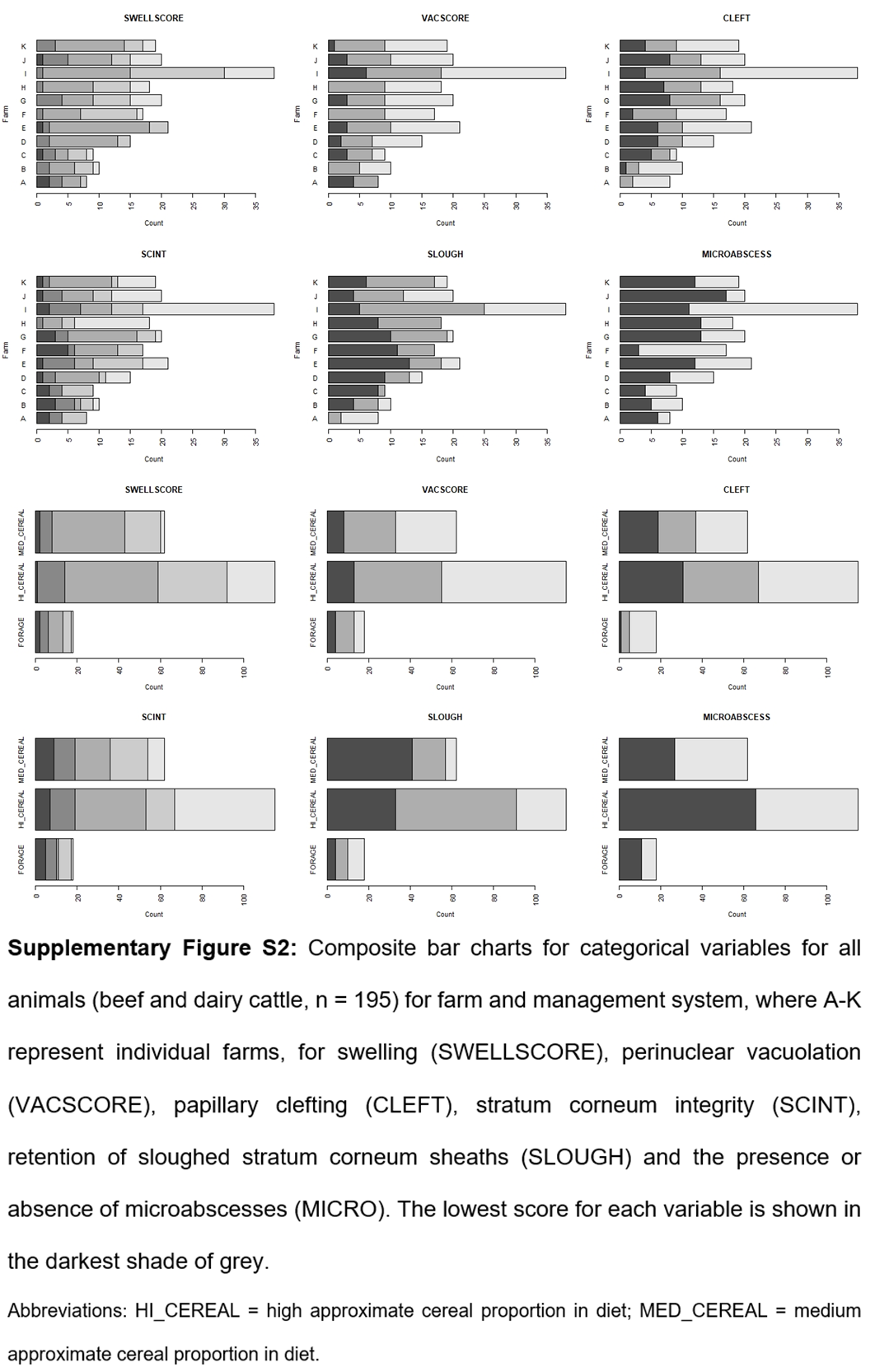


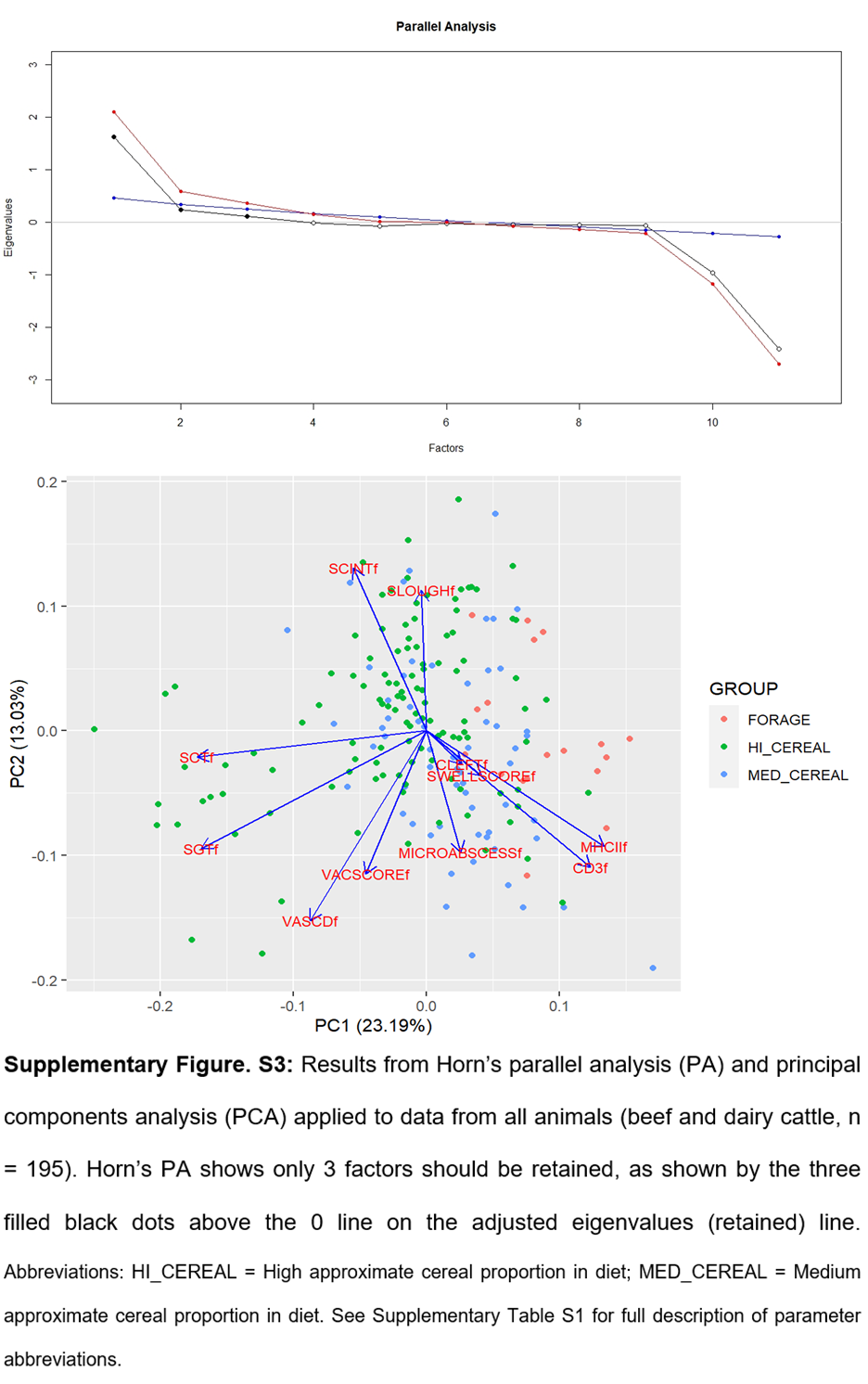


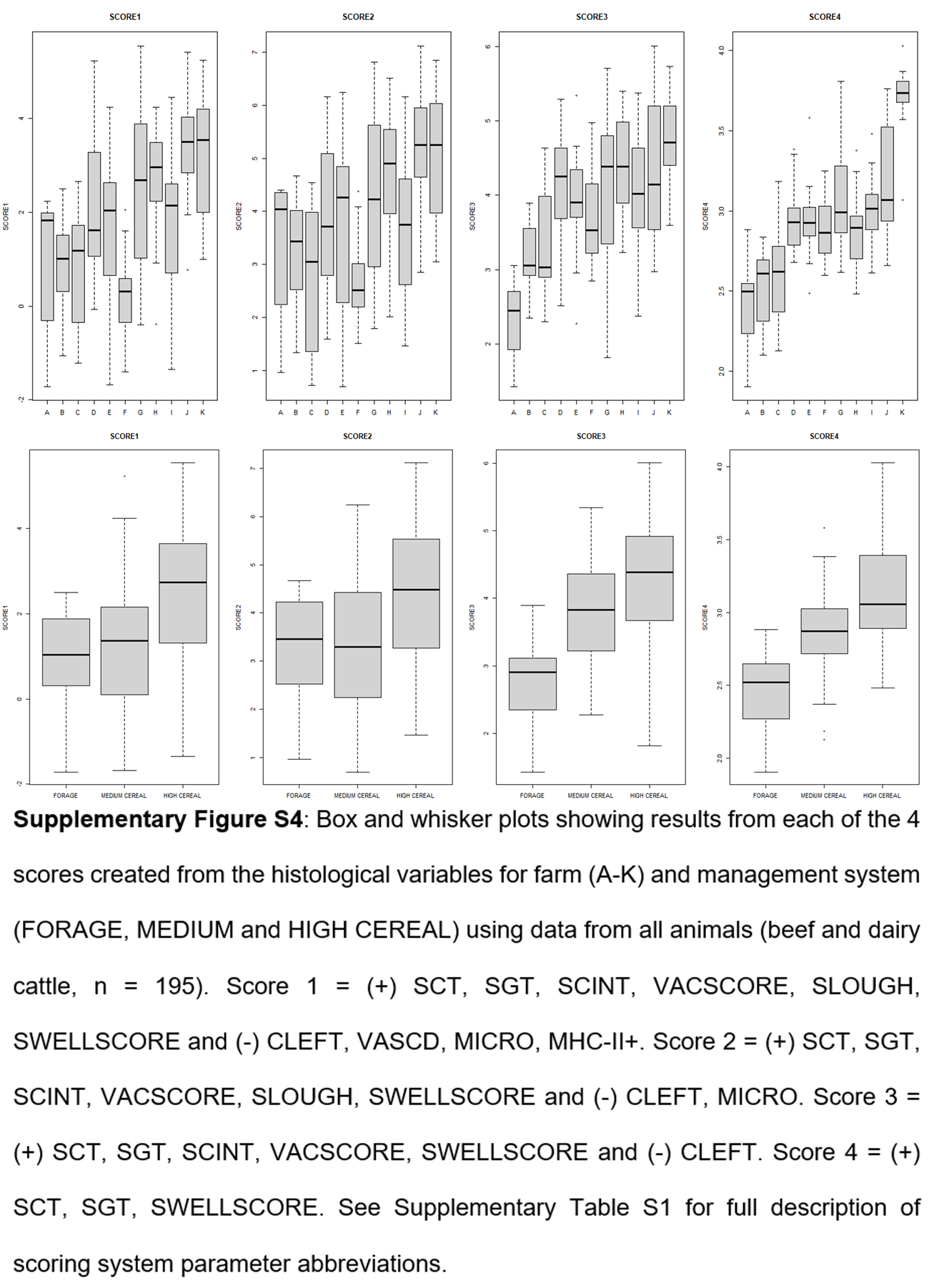


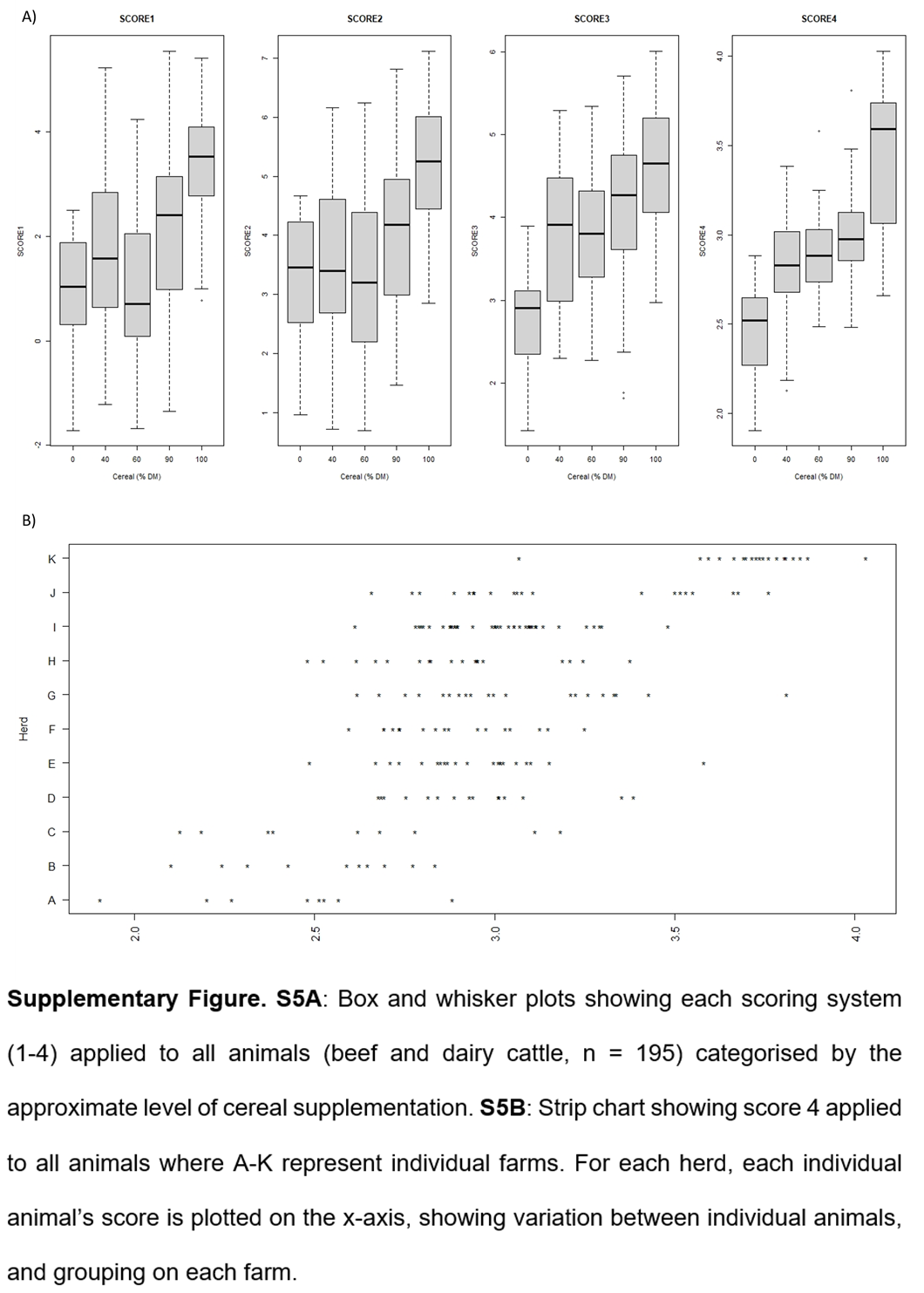


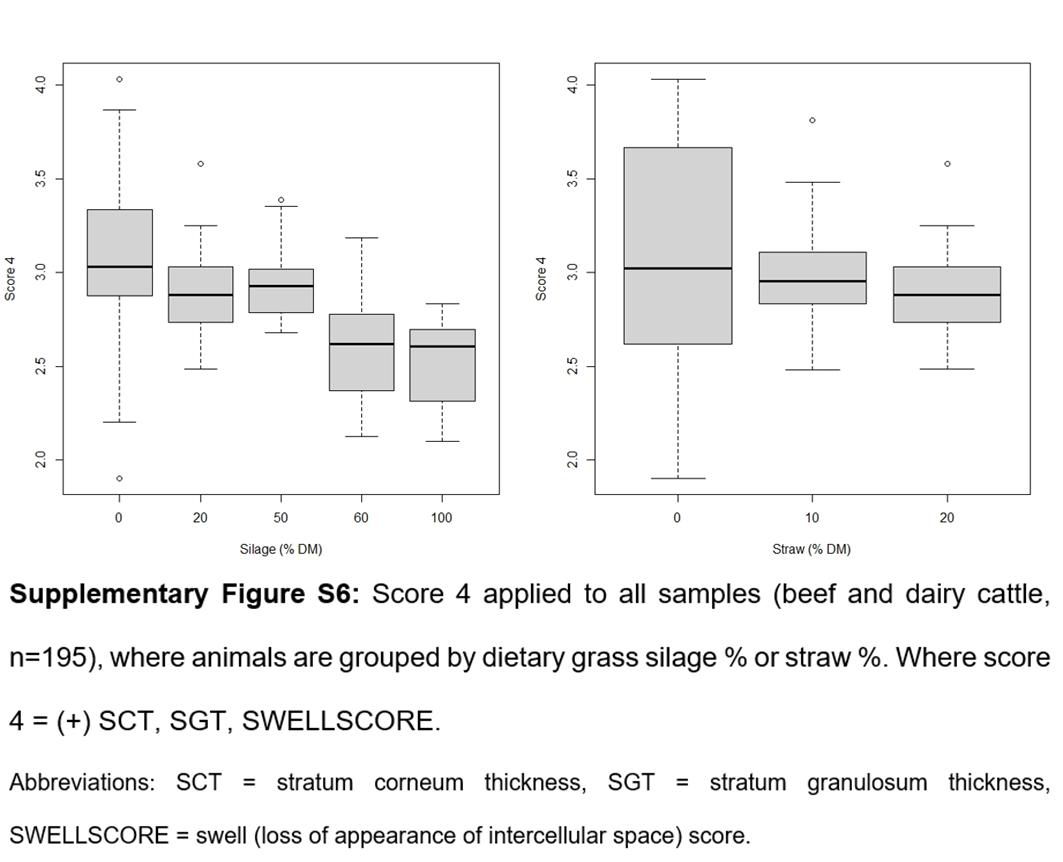

Supplement: Supplementary data 1 [file mmc1.docx]
